# Low molecular weight heparin is useful in adult COVID-19 inpatients. Experience during the first Spanish wave: observational study

**DOI:** 10.1590/1516-3180.2021.0098.r1.08062021

**Published:** 2021-08-13

**Authors:** Jose Ramon Gonzalez-Porras, Moncef Belhassen-Garcia, Amparo Lopez-Bernus, Luis Mario Vaquero-Roncero, Beatriz Rodriguez, Cristina Carbonell, Raul Azibeiro, Alberto Hernandez-Sanchez, Jose Ignacio Martin-Gonzalez, Juan Miguel Manrique, Gloria Alonso-Claudio, Felipe Alvarez-Navia, Jose Ignacio Madruga-Martin, Ronald Paul Macias-Casanova, Jorge García-Criado, Francisco Lozano, Jose Carlos Moyano, Miguel Vicente Sanchez-Hernandez, Víctor Sagredo-Meneses, Rafael Borras, Jose María Bastida, Guillermo Hernández-Pérez, Antonio Javier Chamorro, Miguel Marcos, Jose Angel Martin-Oterino

**Affiliations:** I MD, PhD. Physician, Department of Hematology, Complejo Asistencial Universitario de Salamanca (CAUSA), Instituto de Investigación Biomédica de Salamanca (IBSAL), Universidad de Salamanca (USAL), Salamanca, Spain.; II MD, PhD. Physician, Department of Internal Medicine, Complejo Asistencial Universitario de Salamanca (CAUSA), Instituto de Investigación Biomédica de Salamanca (IBSAL), Universidad de Salamanca (USAL), Salamanca, Spain.; III MD, PhD. Physician, Department of Internal Medicine, Complejo Asistencial Universitario de Salamanca (CAUSA), Instituto de Investigación Biomédica de Salamanca (IBSAL), Universidad de Salamanca (USAL), Salamanca, Spain.; IV MD, PhD. Physician, Department of Anesthesiology and Reanimation, Complejo Asistencial Universitario de Salamanca (CAUSA), Instituto de Investigación Biomédica de Salamanca (IBSAL), Universidad de Salamanca (USAL), Salamanca, Spain.; V MD. Physician, Department of Internal Medicine, Complejo Asistencial Universitario de Salamanca (CAUSA), Instituto de Investigación Biomédica de Salamanca (IBSAL), Universidad de Salamanca (USAL), Salamanca, Spain.; VI MD, PhD. Physician, Department of Internal Medicine, Complejo Asistencial Universitario de Salamanca (CAUSA), Instituto de Investigación Biomédica de Salamanca (IBSAL), Universidad de Salamanca (USAL), Salamanca, Spain.; VII MD. Physician, Department of Hematology, Complejo Asistencial Universitario de Salamanca (CAUSA), Instituto de Investigación Biomédica de Salamanca (IBSAL), Universidad de Salamanca (USAL), Salamanca, Spain.; VIII MD. Physician, Department of Hematology, Complejo Asistencial Universitario de Salamanca (CAUSA), Instituto de Investigación Biomédica de Salamanca (IBSAL), Universidad de Salamanca (USAL), Salamanca, Spain; IX MD, PhD. Physician, Department of Internal Medicine, Complejo Asistencial Universitario de Salamanca (CAUSA), Instituto de Investigación Biomédica de Salamanca (IBSAL), Universidad de Salamanca (USAL), Salamanca, Spain.; X MD. Physician, Department of Internal Medicine, Complejo Asistencial Universitario de Salamanca (CAUSA), Instituto de Investigación Biomédica de Salamanca (IBSAL), Universidad de Salamanca (USAL), Salamanca, Spain.; XI MD. Physician, Department of Internal Medicine, Complejo Asistencial Universitario de Salamanca (CAUSA), Instituto de Investigación Biomédica de Salamanca (IBSAL), Universidad de Salamanca (USAL), Salamanca, Spain.; XII MD. Physician, Department of Internal Medicine, Complejo Asistencial Universitario de Salamanca (CAUSA), Instituto de Investigación Biomédica de Salamanca (IBSAL), Universidad de Salamanca (USAL), Salamanca, Spain.; XIII MD, PhD. Physician, Department of Internal Medicine, Complejo Asistencial Universitario de Salamanca (CAUSA), Instituto de Investigación Biomédica de Salamanca (IBSAL), Universidad de Salamanca (USAL), Salamanca, Spain.; XIV MD. Physician, Department of Internal Medicine, Complejo Asistencial Universitario de Salamanca (CAUSA), Instituto de Investigación Biomédica de Salamanca (IBSAL), Universidad de Salamanca (USAL), Salamanca, Spain.; XV MD. Physician, Department of Emergency, Complejo Asistencial Universitario de Salamanca (CAUSA), Instituto de Investigación Biomédica de Salamanca (IBSAL), Universidad de Salamanca (USAL), Salamanca, Spain.; XVI MD, PhD. Physician, Department of Angiology and Vascular Surgery, Complejo Asistencial Universitario de Salamanca (CAUSA), Instituto de Investigación Biomédica de Salamanca (IBSAL), Universidad de Salamanca (USAL), Salamanca, Spain.; XVII MD, PhD. Physician, Department of Clinical Biochemistry, Complejo Asistencial Universitario de Salamanca (CAUSA), Instituto de Investigación Biomédica de Salamanca (IBSAL), Universidad de Salamanca (USAL), Salamanca, Spain.; XVIII MD. Physician, Department of Anesthesiology and Reanimation, Complejo Asistencial Universitario de Salamanca (CAUSA), Instituto de Investigación Biomédica de Salamanca (IBSAL), Universidad de Salamanca (USAL), Salamanca, Spain.; XIX MD. Physician, Department of Intensive Care Medicine, Complejo Asistencial Universitario de Salamanca (CAUSA), Instituto de Investigación Biomédica de Salamanca (IBSAL), Universidad de Salamanca (USAL), Salamanca, Spain.; XX MD. Physician, Department of Emergency, Complejo Asistencial Universitario de Salamanca (CAUSA), Instituto de Investigación Biomédica de Salamanca (IBSAL), Universidad de Salamanca (USAL), Salamanca, Spain.; XXI MD, PhD. Physician, Department of Hematology, Complejo Asistencial Universitario de Salamanca (CAUSA), Instituto de Investigación Biomédica de Salamanca (IBSAL), Universidad de Salamanca (USAL), Salamanca, Spain.; XXII MD. Physician, Department of Internal Medicine, Complejo Asistencial Universitario de Salamanca (CAUSA), Instituto de Investigación Biomédica de Salamanca (IBSAL), Universidad de Salamanca (USAL), Salamanca, Spain.; XXIII MD, PhD. Physician, Department of Internal Medicine, Complejo Asistencial Universitario de Salamanca (CAUSA), Instituto de Investigación Biomédica de Salamanca (IBSAL), Universidad de Salamanca (USAL), Salamanca, Spain.; XXIV MD, PhD. Physician, Department of Internal Medicine, Complejo Asistencial Universitario de Salamanca (CAUSA), Instituto de Investigación Biomédica de Salamanca (IBSAL), Universidad de Salamanca (USAL), Salamanca, Spain.; xxv MD, PhD. Physician, Department of Internal Medicine, Complejo Asistencial Universitario de Salamanca (CAUSA), Instituto de Investigación Biomédica de Salamanca (IBSAL), Universidad de Salamanca (USAL), Salamanca, Spain.

**Keywords:** COVID-19, SARS-CoV-2, Thrombosis, Pulmonary embolism, Coronavirus disease 2019, Coagulopathy, Bleeding

## Abstract

**BACKGROUND:**

The intensity of the thromboprophylaxis needed as a potential factor for preventing inpatient mortality due to coronavirus disease-19 (COVID-19) remains unclear.

**OBJECTIVE:**

To explore the association between anticoagulation intensity and COVID-19 survival.

**DESIGN AND SETTING:**

Retrospective observational study in a tertiary-level hospital in Spain.

**METHODS:**

Low-molecular-weight heparin (LMWH) status was ascertained based on prescription at admission. To control for immortal time bias, anticoagulant use was analyzed as a time-dependent variable.

**RESULTS:**

690 patients were included (median age, 72 years). LMWH was administered to 615 patients, starting from hospital admission (89.1%). 410 (66.7%) received prophylactic-dose LMWH; 120 (19.5%), therapeutic-dose LMWH; and another 85 (13.8%) who presented respiratory failure, high D-dimer levels (> 3 mg/l) and non-worsening of inflammation markers received prophylaxis of intermediate-dose LMWH. The overall inpatient-mortality rate was 38.5%. The anticoagulant nonuser group presented higher mortality risk than each of the following groups: any LMWH users (HR 2.1; 95% CI: 1.40-3.15); the prophylactic-dose heparin group (HR 2.39; 95% CI, 1.57-3.64); and the users of heparin dose according to biomarkers (HR 6.52; 95% CI, 2.95-14.41). 3.4% of the patients experienced major hemorrhage. 2.8% of the patients developed an episode of thromboembolism.

**CONCLUSIONS:**

This observational study showed that LMWH administered at the time of admission was associated with lower mortality among unselected adult COVID-19 inpatients. The magnitude of the benefit may have been greatest for the intermediate-dose subgroup. Randomized controlled trials to assess the benefit of heparin within different therapeutic regimes for COVID-19 patients are required.

## INTRODUCTION

The disease caused by severe acute respiratory syndrome coronavirus 2 (SARS-CoV-2), known as coronavirus disease-19 (COVID-19), is a new pandemic that appeared in the city of Wuhan, China, in December 2019.^1^^,^^2^ Over the past four months, COVID-19 has become a worldwide pandemic, such that 32,150,495 cases and 982,680 deaths globally have been reported. In Spain, 682,267 cases and 45,252 deaths were reported up to September 24, 2020. Although the majority of COVID-19 cases have resolved spontaneously, some have developed various fatal complications, including organ failure, septic shock, pulmonary edema, severe pneumonia and SARS.^3^

Current data support the concept that disseminated intravascular coagulation (DIC) in sepsis is a coagulation disorder induced by infection, and that it also represents an acute systemic inflammatory response that leads to endothelial dysfunction.^4^^,^^5^ Recent data in the literature show that severe COVID-19 is commonly complicated with coagulopathy and that DIC might exist in the majority of deaths.^6^ Moreover, a remarkably high incidence of venous thromboembolism (VTE) has been reported in patients hospitalized with COVID-19.^7^

Heparin may have positive effects on COVID-19 patients.^8^ The American College of Chest Physicians (ACCP) recommends use of the Padua prediction score, which is a validated risk assessment model, in order to identify hospitalized medical patients who are at high risk of VTE and who should therefore receive thromboprophylaxis during their hospital stay.^9^ However, the substantially high incidence of VTE and overt DIC among COVID-19 patients could justify use of extensive thromboprophylaxis.

Based on these findings, it seems that prophylactic doses of heparin for patients with severe COVID-19 and coagulopathy could be useful, and this has been recommended by some expert consensuses.^8^^,^^10^^–^^12^ Nonetheless, the high incidence of coagulopathy and thrombotic complications that is seen among COVID-19 patients despite use of antithrombotic prophylaxis could be important for decision-making with regard to the intensity of thromboprophylaxis to be applied. Therefore, the benefits of high doses of antithrombotic drugs in COVID-19 cases need to be clarified.

## OBJECTIVE

The present study was designed to explore the intensity of the thromboprophylaxis needed as a potential factor for preventing in-hospital mortality associated to COVID-19.

## METHODS

### Study design and population

We performed a retrospective observational study in Spain on all patients with a diagnosis of COVID-19 who had been hospitalized at the University Hospital of Salamanca between March 1, 2020, and April 7, 2020. The diagnosis of COVID-19 was performed made in accordance with the interim guidance from the World Health Organization. It was then confirmed through detection of the ribonucleic acid (RNA) of SARS-CoV-2 in the microbiological laboratory of the University Hospital of Salamanca.^13^ The only exclusion criterion was age below 18 years. This study was conducted in accordance with the Declaration of Helsinki and was approved by the Ethics Committee of the University Hospital of Salamanca (code: CEIm PI2020-04-472) on April 16, 2020.

### Laboratory procedures and data collection

The baseline characteristics of the patients were retrospectively collected from the electronic medical record system and from the concomitant therapies. We started a registry of patients hospitalized due to COVID 19 in our hospital that was updated every day. A COVID team (acknowledgement section) was in charge of collection of clinical and biological variables. The final outcome (survivor or non-survivor) was also extracted from the medical records. The samples for coagulation tests were collected on admission. Prothrombin time (PT), activated partial thromboplastin time (aPTT), fibrinogen and D-dimer were detected using an ACL TOP 500 CTS coagulation analyzer and original reagents (Werfen Spain SAU, L’Hospitalet de Llobregat, Spain). The DIC-ISTH score was calculated on the basis of the general criteria of the International Society on Thrombosis and Haemostasis (ISTH).^14^

### Heparin prescription

The patients’ low-molecular-weight heparin (LMWH) status was ascertained based on prescription at admission. They were stratified according to the LMWH regimen received, into four groups: non-heparin, prophylactic-dose heparin, therapeutic-dose or heparin dose according to biomarkers.

The prophylactic-dose heparin group was defined as patients who received prophylactic LMWH starting from admission, with prescription in accordance with the Padua VTE risk assessment model.^9^

The prophylactic-dose heparin patients were treated with enoxaparin (40 mg) or bemiparin (3,500 units subcutaneously (SC)) once daily, or if they had a creatinine clearance (CLCr) lower than 30 ml/min upon starting on LMWH, the doses would be enoxaparin (20 mg) or bemiparin (2500 units SC), once daily. Because of warnings about increased thrombotic risk among COVID-19 patients, our local guidelines have endorsed the use of prophylactic-dose heparin as a measure to prevent VTE, for all adult COVID-19 inpatients since March 20, 2020.

Therapeutic-dose heparin, consisting of enoxaparin (1 mg/kg SC bid) or bemiparin (115 IU anti-Xa/kg SC), once daily, was prescribed from the time of admission for patients who were taking oral anticoagulants before admission and who presented very high risk of thrombosis.

From April 30 onwards, we used specified heparin doses for high thrombotic-risk patients (heparin dose according to biomarkers). This cohort comprised patients receiving prophylactic LMWH who presented respiratory failure, high D-dimer levels (> 3 mg/l) and non-worsening of inflammation markers. The per-protocol dimer-D cutoff used was six times greater than the upper limit of normality. This heparin-dose group according to biomarkers presented suspicion of pulmonary embolism, but angiographic computed tomography (CT) did not confirm any presence of pulmonary embolism (PE). This group received enoxaparin (1 mg/kg) or bemiparin (5000 units SC), once daily (intermediate dose of heparin). For any patients with CLCr lower than 30 ml/min, enoxaparin or bemiparin was administered at 0.5 mg/kg or 3500 units SC once daily, respectively.

In the non-heparin group, the patients did not receive any heparin treatment, due to contraindication and/or a low risk of VTE, as shown by the Padua model.

Information on any side effects was also collected from the medical records. Special attention was given to bleeding events: major bleeding was defined as fatal bleeding and/or symptomatic bleeding in a critical area or organ, such as intracranial, intraspinal, intraocular, retroperitoneal, intraarticular or pericardial bleeding, or intramuscular bleeding with compartment syndrome, and/or bleeding causing a fall in hemoglobin level of 2 g/dl or more, or leading to transfusion of two or more units of whole blood or red cells.^15^ VTE was defined as deep venous thrombosis (DVT) diagnosed through ultrasonography, or as pulmonary embolism (PE) diagnosed through helical chest computed tomography (CT) scan. For arterial thrombotic events, ischemic stroke, myocardial infarction and systemic arterial embolism, the World Health Organization definitions were used.

### Statistical analysis

A descriptive statistical analysis was performed after including all the data in an Excel spreadsheet (Microsoft Corp., Redmond, Washington, United States). The normality of distribution of the continuous variables among survivors and non-survivors was evaluated by means of the Kolmogorov-Smirnov test. Continuous variables with normal distribution were presented as the mean (with standard deviation, SD); non-normal variables were reported as the median (with interquartile range, IQR, 25^th^ to 75^th^ percentile). Qualitative values were presented as percentages and absolute numbers.

We used nonparametric tests to compare quantitative variables if the distribution was not normal (Mann-Whitney U test) and parametric tests if it was normal (Student's t test). The Fisher exact test or chi-square test was used for comparison of categorical variables, as appropriate. Cox proportional hazards regression was used to calculate hazard ratios (HRs) and 95% confidence intervals (CIs) for COVID-19 death.

Anticoagulant use after admission was analyzed as a time-dependent variable. The follow-up started at the admission and continued until death or right censoring (June 1, 2020), whichever occurred first. The time metric was days since the baseline. The main analysis was performed by adjusting the Cox regression model for variables with significant statistically differences in the univariate analysis for mortality, to calculate hazard ratios (HRs) and 95% confidence intervals (CIs) for COVID-19 deaths. The analysis was performed separately for: (i) users of any anticoagulant drug compared with nonusers; (ii) users of prophylactic-dose heparin compared with anticoagulant nonusers; (iii) users of therapeutic-dose heparin compared with anticoagulant nonusers; and (iv) users of heparin dose according to biomarkers compared with anticoagulant nonusers. To control for immortal time bias, anticoagulant use was analyzed as a time-dependent variable.

The significance level was set at P < 0.05. The Statistical Package for the Social Sciences 21 software (SPSS; IBM, Chicago, Illinois, United States) and the Stata 15 software (Stata Statistical Software: release 15, 2017; StataCorp LLC, College Station, Texas, United States) were used to perform the statistical analysis.

## RESULTS

A total of 690 consecutive COVID-19 patients admitted to the University Hospital of Salamanca were enrolled. At the time of this analysis (June 1, 2020), 266 patients (38.5%) had died, 422 (61.4%) had been discharged and one (0.1%) remained hospitalized. The inpatient mortality rate was 38.5%. The median age of the study population was 72 years (IQR: 64-85). There were 413 male patients (59.8%) and comorbidities were present in nearly half of the patients (48.9%) (**Table 1**).

**Table 1:** Baseline characteristics and coagulation parameters of COVID-19 patients on admission

		Total (n = 690)	Survivors (n = 424)	Non-survivors (n = 266)	P-value
**Age, mean (± standard deviation)**		72.48 (13.83)	67.17 (13.39)	81.18 (9.43)	< 0.001
**Sex, male/female, n**		416/274	253/174	163/100	0.477
**Pneumonia, n (%)**		422 (61.2)	269 (63.0)	153 (58.2)	0.207
**BMI > 30, n (%)**		146 (26.1)	100 (28.1)	46 (22.7)	0.147
**Charlson comorbidity index ≥ 1, n (%)**		428 (62.3)	218 (51.3)	210 (80.2)	< 0.001
**On admission**					
	PT (sec), median (IQR)	12.9 (11.5-14.8)	12.6 (11.4-14.2)	13.5 (11.6-16.8)	<0.001
	aPTT (sec), median (IQR)	33.6 (30.8-36.9)	33.6 (31.5-36.6)	33.76(30.5- 37.4)	0.762
	Fibrinogen (mg/dl), median (IQR)	637 (504-796)	619 (493-769)	666 (529-808)	0.138
	D-dimer (g/l), median (IQR)	0.8 (0.5-1.70)	0.7 (0.4-1.1)	1.3 (0.8-3.17)	< 0.001
	Platelets (x10^9^/l), median (IQR)	186 (144-244)	187 (146-250)	1813(141-232)	0.314
	Lymphocytes (x10^9^/l), median (IQR)	0.88 (0.64-1.23)	0.95 (0.70-1.27)	0.74 (0.55-1.13)	0.001
	LDH (U/l), median (IQR)	358 (286-458)	338 (276-425)	403 (313-530)	< 0.001
	DIC-ISTH score, median (IQR)	2 (0-2)	2 (0-2)	2 (2-3)	< 0.001
**Treatments**					
	Heparin, n (%)	615 (89.1)	400 (93.7)	215 (81.7)	< 0.001
	Corticosteroids, n (%)	368 (53.3)	219 (51.3)	149 (56.7)	0.170
	Hydroxychloroquine, n (%)	645(93.9)	416 (98.1)	229 (87.1)	< 0.001
	Lopinavir/ritonavir, n (%)	581 (84.2)	388 (91.5)	193 (73.0)	< 0.001
	Tocilizumab, n (%)	216 (31.3)	158 (37.0)	58 (22.1)	< 0.001

BMI = body mass index; PT = prothrombin time; aPTT = activated partial thromboplastin time; IQR = interquartile range; LDH = lactate dehydrogenase; DIC = disseminated intravascular coagulation; ISTH = International Society on Thrombosis and Haemostasis.

Normal ranges: PT (11.1 – 15.8 seconds); aPTT (27- 40 seconds); fibrinogen (130-400 mg/dl); D-dimer (< 0.5 g/l); platelet count (150×10^9^/l – 400×10^9^/l); lymphocyte count (1.2-3.5x10^9^/l); LDH (135-225).

The treatment for COVID-19 was not homogeneous and changed over time in accordance with the national and international recommendations: 341 patients (49.4%) received corticosteroids, 439 (63.6%) received hydroxychloroquine and 388 (56.2%) received lopinavir/ritonavir. Tocilizumab, to manage cytokine storm syndrome, was administered to 207 (30%).

Heparin was administered to 615 patients from the time of hospital admission (89.1%). The median time on treatment with LMWH was 14 days (IQR ± 8). 75 patients (10.8%) did not receive any heparin.

### Baseline characteristics of the patients and features predicting survival (comparison between survivors and non-survivors)

The survivors were significantly younger (median: 67 years) versus non-survivors (81 years) (P < 0.001). Patients with Charlson comorbidity index ≥ 1 were statistically more frequently non-survivors than survivors (80.2% versus 51.3%; P < 0.001). In addition, non-survivors presented higher D-dimer levels (1.3 mg/l versus 0.7 mg/l; P < 0.001) and longer PT (13.5 sec versus 12.7 sec; P = 0.001) than survivors. The DIC-ISTH score [2 (0-2) versus 2 (2-3)] was quite similar between the groups. The treatment with LMWH was associated with a lower inpatient mortality rate (**Table 1**).

### Baseline characteristics, treatment received, UCI admission and mortality among the patients (comparison between heparin subgroups)

Out of the 615 patients who received heparin, 410 (66.7%) received a prophylactic dose, 120 (19.5%) received a therapeutic dose and 85 other patients (13.8%) undergoing LMWH prophylaxis presented respiratory failure, high D-dimer levels (> 3 mg/l) and non-worsening of inflammation markers, and thus received an intermediate heparin dose (heparin-dose group according to biomarkers). **Table 2** shows the baseline characteristics, treatments received, intensive care unit (ICU) admission and mortality according to heparin group.

**Table 2:** Baseline characteristics and coagulation parameters of COVID-19 patients according to heparin group

		Non-heparin (n = 75)	Prophylactic-dose heparin (n = 410)	Therapeutic-dose heparin (n = 120)	Heparin dose according to biomarkers (n = 85)	P-value
**Age, mean (± standard deviation)**		75.2(15.5)	71.7 (14.1)	76.3 (11.2)	67.7 (11.9)	0.004
**Sex, male/female, n**		46/29	239/171	71/49	60/25	0.208
**BMI, mean (± standard deviation)**		29.4 (4.9)	28.9 (5.3)	28.8 (4.8)	30.4 (6.5)	0.911
**Pneumonia, n (%)**		40 (53.3)	255(62.2)	74 (61.7)	53 (62.4)	0.535
**Charlson comorbidity index > 1, n (%)**		51 (69.9)	241 (58.8)	87 (73.1)	49 (57.6)	0.014
**On admission**						
	PT (sec), median (IQR)	13.3 (11.4-15.6)	12.4 (11.4-13.8)	16.1 (12.4-23.6)	13.2 (11.8-15.8)	< 0.001
	aPTT (sec), median (IQR)	33.5 (30.1-36.6)	33.2 (30.9-35.4)	34.1 (30.3- 38.1)	36.5 (32.9-42.1)	< 0.001
	Fibrinogen (mg/dl), median (IQR)	571 (498-721)	637 (493-796)	621 (532-769)	708 (538-832)	0.052
	D-dimer (g/l), median (IQR)	1.1 (0.5-3.4)	0.8 (0.5-1.6)	0.75 (0.4-1.3)	0.9 (0.5- 2.2)	0.101
	Platelets (x10^9^/l), median (IQR)	167 (124-232)	191 (146-253)	179 (144-235)	180 (140-238)	0.034
	Lymphocytes (x10^9^/l), median (IQR)	0.91 (0.59-1.28)	0.94 (0.66-1.28)	0.75 (0.61-1.04)	0.84 (0.61-1.11)	0.017
	LDH (U/l), median (IQR)	353 (277-484)	351 (278-441)	364 (286-476)	392 (307-512)	0.019
	DIC score, median (IQR)	2 (2-3)	2 (0-2)	2 (2-3)	2 (0-3)	< 0.001
**Treatments**				Total 126		
	Corticosteroids, n (%)	22 (29.3)	208 (50.7)	84 (70)	54 (63)	<0.001
	Hydroxychloroquine, n (%)	57 (76.0)	390 (95.1)	115 (96.6)	83 (100)	< 0.001
	Lopinavir/ritonavir, n (%)	50 (66.7)	3467 (84.6)	105 (88.2)	78 (94-0)	< 0.001
	Tocilizumab, n (%)	9 (12)	116 (28.3)	36 (20.0)	55 (64.7)	< 0.001
**ICU admission, n (%)**		5 (6.7)	21 (5.1)	13 (10.8)	41 (48.2)	< 0.001
**Death, n (%)**		48 (64.0)	134 (32.7)	57 (47.5)	24 (28.2)	< 0.001

BMI = body mass index; PT = prothrombin time; aPTT = activated partial thromboplastin time; IQR = interquartile range; LDH = lactate dehydrogenase; DIC = disseminated intravascular coagulation; ISTH = International Society on Thrombosis and Haemostasis; ICU = intensive care unit.

Normal range: PT (11.1-15.8 seconds); aPTT (27-40 seconds); fibrinogen (130-400 mg/dl); D-dimer (< 0.5 g/l); platelet count (150×10^9^/l-400×10^9^/l); lymphocyte count (1.2-3.5x10^9^/l); LDH (135-225).

There were statistically significant differences among the four heparin groups regarding age, comorbidities, prothrombin time, aPTT time, platelet count, lymphocyte count, lactate dehydrogenase (LDH) levels and disseminated intravascular coagulation-International Society on Thrombosis and Haemostasis (DIC-ISTH) scores on admission. The younger patients with fewer comorbidities were more likely to be in the heparin-dose group according to biomarkers. The gender, body mass index (BMI), presence of pneumonia and levels of fibrinogen and D-dimer at diagnosis were similar in all the heparin groups. The percentage of intensive care unit (ICU) admission was higher in the heparin-dose group according to biomarkers. The inpatient mortality was lower in the heparin-dose group according to biomarkers (28.2%) and the prophylactic-dose group (32.7%).

### COVID-19 survival in relation to use of heparin

**Figure 1:**
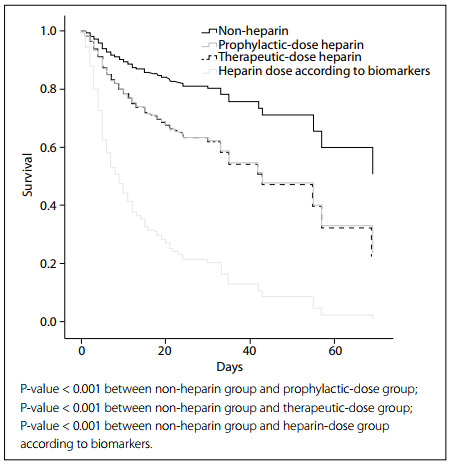
Overall survival based on type of heparin use.

**Figure 1** shows the overall survival based on type of heparin use. The anticoagulant nonuser group presented higher mortality risk than any LMWH users (HR 2.1; 95% CI: 1.40-3.15). Three other variables retained their independent prognostic value for predicting higher inpatient mortality: age, DIC-ISTH score and LDH levels. **Table 3** shows the results subdivided according to the use of different heparin doses.

**Table 3:** Cox regressions. All heparin users compared with nonusers, prophylactic-dose heparin users compared with anticoagulant nonusers, therapeutic-dose heparin users compared with anticoagulant nonusers and heparin-dose users according to biomarkers compared with anticoagulant nonusers. Anticoagulant use was analyzed as a time-dependent variable

		Multivariable-adjusted hazard ratios*
**All anticoagulant users compared with nonusers**		
	None	2.10 (1.40-3.15)
	Any	Ref
**Age**		1.89 (1.62-2.20)
**DIC-ISTH score**		1.23 ()1.07-1.41)
**LDH level**		1.00 (1.00-1.00)
**Prophylactic-dose heparin users compared with anticoagulant nonusers**		
	None	2.39 (1.57-3.64)
	Prophylactic-dose heparin	Ref
**Age**		2.05 (1.71-2.46)
**Charlson comorbidity index**		1.79 (1.10-2.93)
**LDH levels**		1.00 (1.00-1.00)
**Therapeutic-dose heparin users compared with anticoagulant nonusers**		
	None	2.69 (1.61-4.50)
	Therapeutic-dose heparin	Ref
**Age**		1.73 (1.33-2.17)
**DIC-ISTH score**		1.22 (1.00-1.50)
**LDH levels**		1.00 (1.001-1.00)
**Heparin-dose users according to biomarkers compared with anticoagulant nonusers**		
	None	6.52 (2.95-14.64)
	Heparin dose according to biomarkers	Ref
**Age**		1.65 (1.22-2-23)
**LDH levels**		1.00 (1.00-1-00)

* Multivariable-adjusted hazard ratios (with 95% confidence intervals) in relation to deaths. Adjusted variables are age, Charlson comorbidity Index, DIC-ISTH score, LDH, lymphocytes, prothrombin time and treatments.

DIC = disseminated intravascular coagulation; ISTH = International Society on Thrombosis and Haemostasis; LDH =lactate dehydrogenase.

The anticoagulant nonuser group also presented higher mortality risk than the prophylactic-dose heparin group (HR 2.39; 95% CI, 1.57-3.64). According to this model, the other mortality risk factors were age, Charlson comorbidity index and LDH levels.

The anticoagulant nonusers were at significantly higher risk of COVID-19 death than were the therapeutic-dose LMWH users (HR 2.69; 95% CI, 1.61–4.50). Age, DIC-ISTH scores and LDH levels were the other mortality risk factors.

Lastly, the anticoagulant nonuser group presented higher mortality risk than the users of heparin dose according to biomarkers (HR 6.52; 95% CI, 2.95-14.41).

### Bleeding and thromboembolic complications

Among the 690 patients, 24 patients (3.4%) experienced major hemorrhage, but only one case was fatal (**Table 4**). Two cases of major bleeding complications occurred in patients without heparin (2.6%), eight cases of major hemorrhage occurred in the low-heparin-dose group (1.9%), six cases of major bleeding complications occurred among the patients with therapeutic-dose heparin (5%) and eight cases of major bleeding occurred in the heparin-dose group according to biomarkers (9.4%) (P = 0.007, between heparin groups). Nineteen patients (2.8%) developed an episode of thromboembolism, which was fatal in three cases (**Table 5**).

**Table 4:** Major hemorrhage events in COVID-19 patients

Site of bleeding	Heparin group	Days after starting use of heparin	Criterion for defining event as major bleeding	Fatal
Intracranial	Dose according to biomarkers	1	Critical organ	No
Intracranial	Non-heparin	19*	Critical organ	Yes
Lung	Dose according to biomarkers	3	Transfusion of 2 units of RBCs	No
Lung	Prophylactic-dose	5	Transfusion of 2 units of RBCs	No
Gastrointestinal	Non-heparin	7*	Transfusion of 8 units of RBCs	No
Gastrointestinal	Prophylactic-dose	5	Transfusion of 4 units of RBCs	No
Gastrointestinal	Prophylactic-dose	7	Transfusion of 2 units of RBCs	No
Gastrointestinal	Prophylactic-dose	5	Transfusion of 2 units of RBCs	No
Gastrointestinal	Prophylactic-dose	9	Transfusion of 2 units of RBCs	No
Gastrointestinal	Prophylactic-dose	12	Transfusion of 2 units of RBCs	No
Gastrointestinal	Dose according to biomarkers	6	Transfusion of 2 units of RBCs	No
Gastrointestinal	Therapeutic-dose	9	Transfusion of 2 units of RBCs	No
Gastrointestinal	Prophylactic-dose	3	Transfusion of 2 units of RBCs	No
Gastrointestinal	Therapeutic-dose	13	Fall in Hb level of 3 g/dl	No
Gastrointestinal	Therapeutic-dose	12	Fall in Hb level of 2 g/dl	No
Tracheostomy	Dose according to biomarkers	7	Transfusion of 2 units of RBCs	No
Tracheostomy	Dose according to biomarkers	9	Transfusion of 2 units of RBCs	No
Tracheostomy	Dose according to biomarkers	6	Transfusion of 2 units of RBCs	No
Genitourinary	Prophylactic-dose	5	Fall in Hb level of 3 g/dl	No
Genitourinary	Therapeutic-dose	14	Fall in Hb level of 2 g/dl	No
Genitourinary	Therapeutic-dose	10	Fall in Hb level of 2 g/dl	No
Chest wall hematoma	Dose according to biomarkers	9	Fall in Hb level of 3 g/dl	No
Hematoma catheter size	Dose according to biomarkers	14	Transfusion of 6 units of RBCs	No
Hematoma catheter size	Therapeutic-dose	18	Fall in Hb level of 3 g/dl	No

* In this situation (non-heparin treatment), days after inpatient admission.

**Table 5:** Thromboembolic events in COVID-19 patients

Type of event	Heparin group	Cardiovascular risk factors	Days after starting treatment	Fatal
Pulmonary embolism	Therapeutic-dose	88 years, male, hypertension, dyslipidemia, stroke	1	No
Pulmonary embolism	Dose according to biomarkers	74 years, male, hypertension, dyslipidemia	10	No
Pulmonary embolism	Dose according to biomarkers	56 years, male	22	No
Pulmonary embolism	Therapeutic-dose	74, male, hypertension, atrial fibrillation, rheumatoid arthritis	1	No
Pulmonary embolism	Prophylactic-dose	64 years, female, asthma	6	No
Pulmonary embolism	Prophylactic-dose	73 years, female,	45	No
Pulmonary embolism	Dose according to biomarkers	63 years, male, hypertension, obesity	6	No
Pulmonary embolism	Dose according to biomarkers	66 years, male	8	No
Pulmonary embolism	Prophylactic-dose	82 years, female, hypertension, diabetes	9	No
Deep venous thrombosis	Prophylactic-dose	64 years, male, dyslipidemia	12	No
Deep venous thrombosis	Prophylactic-dose	74 years, female	1	No
Deep venous thrombosis	Dose according to biomarkers	47 years, female, catheter	6	No
Portal thrombosis	Dose according to biomarkers	83 years, female, hypertension, diabetes, dyslipidemia, gallbladder cancer in 2018	2	No
Stroke	Prophylactic-dose	62 years, female, dyslipidemia	16	No
Stroke	Therapeutic-dose	84 years, male, atrial fibrillation,	2	Yes
Myocardial infarction	Dose according to biomarkers	85 years, male, hypertension, diabetes, prior myocardial infarction	3	Yes
Myocardial infarction	Dose according to biomarkers	81 years, male, hypertension, diabetes, dyslipidemia, prior myocardial infarction	2	No
Myocardial infarction	Non-heparin	93 years, female, hypertension	2*	Yes
Critical limb ischemia	Prophylactic-dose	64 years, male, hypertension, diabetes, smoking	10	No

* In this situation (non-heparin treatment), days after inpatient admission.

## DISCUSSION

We report in this retrospective observational study how the administration of LMWH at the time of admission was associated with a reduced mortality rate among unselected adult COVID-19 patients. The magnitude of the benefit may have been greatest for the group of patients who received a heparin dose according to biomarkers. It should be noted that overall, although major bleeding was more frequently reported in the higher dose groups, only one fatal event was reported. In addition, young patients with no comorbidities, low LDH levels and low DIC-ISTH scores at the time of admission presented a significantly lower risk of inpatient mortality.

Overall, infection is a common cause of disseminated intravascular coagulation. Inflammation, infection and other factors can lead to excessive suppression of fibrinolysis and a disrupted anticoagulant system.^16^ Previous reports have observed that COVID-19 patients with severe pneumonia may develop significant abnormalities of coagulation features, DIC and ischemic changes in different tissues. In fact, DIC appeared in most of the deaths in those reports.^6^ SARS-CoV-2 can hyperactivate the innate immune system in excess, thereby causing cytokine storms and damage to the microvascular system and activating coagulation and fibrinolysis. Interleukin-6 (IL-6) is a key factor in the inflammatory factor storm induced by SARS-CoV-2.^17^^,^^18^ On the other hand, ischemia and hypoxia reperfusion injury may contribute to the hypercoagulable state. In this regard, early recognition of COVID-19-associated coagulopathy could be very helpful in anticipating and dealing with the outcomes.

There is no strong evidence to support the idea that routine anticoagulation therapy would be effective for preventing sepsis.^19^ A meta-analysis on randomized controlled trials comparing LMWH versus placebo in sepsis suggested that LMWH might reduce mortality among septic patients.^20^ Another recent meta-analysis suggested that anticoagulation therapy would be beneficial only for patients with sepsis-induced DIC and not for the entire population of patients with sepsis.^21^ Moreover, the guidance for diagnosis and treatment of DIC provided by the ISTH states that use of therapeutic doses of heparin should be considered in cases of DIC in which thrombosis is predominant.^22^ A multicenter cohort study conducted by Japanese institutions reported that use of high-intensity anticoagulation therapy was associated with better outcomes among patients with sepsis-induced DIC.^23^

Currently, there is little information on the use of LMWH in relation to COVID-19. Anticoagulant therapy that was implemented mainly using LMWH at a prophylactic dose was associated with a better prognosis in a series of COVID-19 patients in China, but the infection level was severe in all patients.^8^ However, in our study, we show how the use of a prophylactic dose of LMWH starting from the time of admission to the hospital significantly reduced the inpatient mortality rate among all adult COVID-19 patients. Our findings can possibly be explained by the differences in ethnicity, age (our median age was 72 years, versus 65 years in the Chinese population) and sample size. Our results are in line with expert opinion, which recommends the use of prophylactic LMWH in hospitalized COVID-19 patients.^11^ However, in addition, we suggest that the intensity of the thromboprophylaxis used may be a potential factor for preventing in-hospital mortality associated with COVID-19.

Besides its use as an anticoagulant, heparin has demonstrated excellent anti-inflammatory properties in animal models and clinical trials.^24^ Use of LMWH was found to reduce serum IL-6 levels, which are a key factor in patients with severe COVID-19, and to reduce TNF-α levels.^25^ Heparin has been seen to exert an inhibitory effect on replication activity and against attachment and entry of enveloped viruses, in relation to several viruses: human herpes simplex virus (HSV), human immunodeficiency virus (HIV), SARS coronavirus and influenza virus (H5N1).^26^ Moreover, heparin prevents Zika virus-induced cell death of human neural progenitor cells.^27^ Therefore, the potential anti-inflammatory and antiviral properties of LMWH might partly explain its beneficial mechanism.

Thromboprophylaxis using high doses of LMWH may lead to bleeding, which can be fatal. In our series, major bleeding was presented in 3.4% of the patients and the bleeding rate was significantly higher in the high-heparin-dose group (7.3%). The incidence of major bleeding in critically ill patients who received LMWH prophylaxis was reported to range from 1.2% to 5.4% in three trials.

The rate of thrombosis in our series seemed very low (2.8%). The exact prevalence or incidence of venous thromboembolism in COVID-19 patients is unknown. Different reports have indicated VTE rates ranging from 11% to 31%, and the highest incidence of VTE has been found among patients admitted to intensive care units.^28^

Thrombotic complications have only rarely been described in COVID-19 patients. Klok et al. recently reported that the cumulative incidence of thrombotic complications among ICU patients was 31%.^7^ This cumulative incidence is remarkably high, and was in spite of the finding that all the patients had received at least standard doses of thromboprophylaxis. Those authors emphasized the recommendation to strictly apply pharmacological thrombosis prophylaxis to all COVID-19 patients admitted to an ICU, and strongly suggested that the level of prophylaxis should be increased towards high prophylactic doses.^7^ In another study among hospitalized patients with COVID-19, the overall estimated pooled incidence of VTE was 17.0%.^29^

In our cohort, the percentage of ICU patients with VTE was only 11%. In addition, our use of higher doses of LMWH in a high percentage of patients could explain our low incidence of VTE. Our findings stress the need for exploring the optimal dose of LMWH among COVID-19 patients. In this setting, the hypothesis supporting the notion that high doses of anticoagulants will reduce the risk of thrombosis, DIC and mortality, compared with low doses of anticoagulants, in patients with COVID-19 infection, will be explored in several randomized clinical trials.^30^ Recent real-world data have shown that early starting of prophylactic anticoagulation, compared with no anticoagulation, among patients admitted to hospital with COVID-19, was associated with a decreased risk of 30-day mortality and no increased risk of serious bleeding events.^31^ In addition, in a recent press release dated January 22, 2021, from the United States National Institutes of Health (NIH), which is coordinating a multiplatform randomized controlled trial (RCT), it was reported that therapeutic-dose anticoagulation had been found to be beneficial for decreasing the need for organ support among patients who did not require ICU-level care when they entered the study, regardless of D-dimer level, with a trend toward less mortality.^32^

Increased D-dimer levels in patients with severe COVID-19 have commonly been reported to be a predictor for a dismal outcome. Several authors have observed that patients with severe COVID-19 presented D-dimer levels that were 2.5 to 5-fold higher than those in patients without this.^6^^,^^30^^–^^35^ Zhou et al. found an association between higher D-dimer levels (9-fold higher) and mortality among patients with severe COVID-19.^34^ The risk of severe illness was more frequent in patients with D-dimer levels above 0.5 mg/l.^33^ A pooled analysis on four retrospective observational studies found that D-dimer levels were considerably higher in COVID-19 patients with severe disease than in those without this (weighted mean difference: 2.97 mg/l; 95% CI: 2.47-3.46 mg/l), but the heterogeneity across the four studies was relatively high (i.e. I^2^ = 94%; P < 0.001).^36^ Petrilli et al. showed that there was a relationship between D-dimer level and its trajectory and the frequency of adverse clinical events.^37^ In our unselected cohort of COVID-19 patients, the median D-dimer level on admission was significantly higher in non-survivors (2.1 g/l) than in survivors (0.9 g/l), in the univariate analysis, but the prognostic impact of this finding was not maintained in the multivariate analysis. According to our model, the DIC-ISTH score, which includes D-dimer data, is a more valuable criterion with independent prognostic value for predicting inpatient mortality risk.

In addition, the mortality risk index also included age, LDH, underlying diseases, DIC-ISTH score and use of LMWH, which would facilitate identification of patients with high mortality risk, among unselected adult COVID-19 cases at the time of hospital admission. In fact, the reported area under the receiver operating characteristic curve for this category is of great value (COVID-19 mortality index 0.869).

The benefit of heparin doses needs to be balanced against the risk of bleeding. We observed an excess of bleeding complications in patients who received the highest heparin dose. Along the same lines, bleeding events were observed in another study in 7.8% of the patients hospitalized with COVID-19 and were sensitive to use of escalated doses of anticoagulants and to the nature of data collection.^29^

The limitations of our study are those that are inherent to an observational retrospective single-center study. Potential selection and immortal time bias do exist in this kind of study. Through assessing the potential role and magnitude of this confounding, the inherent differences between the heparin groups can be understood. We had detailed information on patient characteristics among the heparin groups. The analyses were adjusted for multiple background variables to minimize bias. The outcome was survival at the time of the analysis: at that time, only one patient was still hospitalized. On the other hand, to control for immortal time bias, the anticoagulant dose was analyzed as a time-dependent variable.

Although our study focused on coagulation parameters, other variables could also impact on mortality. The concomitant therapy, including LMWH, was not assessed in relation to a control. The true rate of VTE was also perhaps underestimated due to the impossibility of carrying out imaging studies on some patients with clinically suspected VTE. Nonetheless, our report describes the experience of a single center with a large patient population that was homogeneously managed in accordance with the local guidelines, which were regularly updated with the emerging information. If a multicenter study had been conducted, this might have given rise to introduction of additional confounding factors, due to the heterogeneity of management protocols across the centers.

## CONCLUSIONS

Our results suggest that application of LMWH at the time of admission significantly reduced the mortality rate among these unselected adult COVID-19 inpatients. The LMWH dose could have prognostic impact, although overall, major bleeding was more frequently reported in the high-dose group. Further research is needed to tailor heparin prophylaxis and ascertain the correct dose for adults COVID-19 patients.
